# Integrating 13 Microarrays to Construct a 6 RNA-binding proteins Prognostic Signature for Gastric Cancer patients

**DOI:** 10.7150/jca.57225

**Published:** 2021-06-11

**Authors:** Liqiang Zhou, Qi Zhou, You Wu, Lin Xin

**Affiliations:** Department of General Surgery, The Second Affiliated Hospital of Nanchang University, Nanchang 330006, Jiangxi, China.

**Keywords:** gastric cancer, RNA-binding protein, prognosis, signature, nomogram, survival

## Abstract

**Background:** It has been confirmed in many tumors that RNA-binding proteins (RBPs) will affect the progress of cancer, but there is still a lack of large-scale research in gastric cancer (GC).

**Methods:** We obtained 13 microarray mRNA expression profiles of the GPL570 platform, and extracted expression from them after integration to analyze the expression differences of RBPs. Enrichment analysis studies the role of these RBPs in GC. Univariate, Lasso and multivariate Cox regression analysis are used to identify independent prognostic hub RBPs, thereby constructing and verifying a prognostic signature. External data and rt-PCR verified the expression of hub RBPs.

**Results:** We have identified 51 dysregulated RBPs in GC. Enrichment analysis shows that it can mainly participate in RNA decomposition, modification, processing, etc. and affect the progress of GC. After multiple statistical analysis, six independent prognostic RBPs of GC were determined and a prognostic signature was developed. According to the median risk value, the training cohort was divided into high-risk and low-risk groups. Considering the clinical characteristics, in training, testing, and complete cohorts, the overall survival rate of the high-risk group was significantly lower than that of the low-risk group, which was confirmed by the time-dependent receiver operating characteristic curve. Univariate and multivariate Cox regression analysis of independent prognostic ability of risk score. In addition, we constructed and verified a nomogram based on the prognostic signature, showing accurate prediction performance. rt-PCR and external data verification are consistent with our conclusions.

**Conclusion:** This study analyzed the overall expression of RPBs in GC and explored its mechanism. A new prognostic signature was developed and verified. A nomogram has also been established and verified, which helps to improve the treatment strategy for GC.

## Introduction

Gastric cancer (GC) is a malignant tumor that originates from epithelial cells of the gastric mucosa. The risk factors include: race, environment, Helicobacter pylori infection, high-salt diet, moldy food, obesity, smoking and other factors. According to the latest statistics, there are 1,033,701 new cases of GC and 782,685 deaths each year, accounting for 5.7% and 8.2% of all tumors, respectively 1. Reports in 2010 showed that the incidence of gastric cancer in China accounted for as high as 42.6% of the world, and it continues to increase 2. This is because GC only shows upper abdominal discomfort in the early stage, belching and other symptoms are similar to gastritis, gastric ulcer and other chronic gastric diseases 1, 3. There are no other specific symptoms, which makes it easy to be ignored, and it is mostly in the late stage when discovered. At present, the main ways to treat GC include: surgery, chemotherapy, and molecular targeted therapy. However, these treatments have the characteristics of easy relapse and drug resistance, which make the 5-year survival rate of GC less than 30% 4. Due to the late detection of GC, the prognosis is poor. Therefore, it is particularly important to find biomarkers that can accurately predict GC.

RNA-binding proteins are critical regulators of transcriptional and posttranscriptional gene expression possessing multiple biological functions. There are highly species-conservative and play a key role in maintaining homeostasis of gene expression 5, 6. Multiple evidences suggest that RBPs is involved in various important cellular processes, such as cell transport, localization, development, differentiation and metabolism. In addition, RBP participates in almost every step of post-transcriptional regulation, supervises the formation and function of transcripts, and maintains cell homeostasis, such as RNA shearing, transport, sequence editing, intracellular localization, and translation control by identifying special RNA binding domains and RNA interactions 7, 8. At present, 1,542 cancer-related RBPs genes have been identified in cancer cells by RNA-seq screening technology 8. More and more studies have shown that RNA modification mediated by RBPs plays a crucial role in the origin and progression of cancer 9, 10. They greatly changed the growth and proliferation of tumor cells, avoided immune surveillance, induced angiogenesis and activated metastasis 11. It has been observed that the expression of RBPs in multiple tumors is dysregulated, and regulate the function of oncogene or tumor suppressor gene 12-15. Therefore, by revealing the basic mechanism of RBP expression and its potential functions, it helps to find new cancer treatment targets and provide new ideas or methods.

In GC, some reports indicate that the expression of RBPs is unregulated and causes the abnormal expression of its target proteins, and these target proteins are closely related to the prognosis of GC. For example, RBFOX3 is highly expressed in GC and predicts a poor prognosis, which can promote the growth and progression of GC by binding to AP-2β to activate the HTERT signal 16. In GC stem cells, Lin28B can maintain cell stemness by binding to NRP-1 to activate Wnt/β-catenin signal transduction 17. In addition, PTBP3 can mediate CAV1 alternative splicing to promote GC metastasis 18. The above studies suggest that RBPs play an important role in GC, which gives us a preliminary understanding of RBPs in GC. However, there is no report on the overall analysis of research in GC. Therefore, we downloaded 13 microarray GC and healthy tissue expression data from Gene Expression Omnibus (GEO) on the GPL570 platform, and completely analyzed the abnormal expression of RBPs between tumor samples and normal samples. Systematically explored their potential functions and molecular mechanisms. Combined with clinical data, these differentially expressed RBPs are screened for genes with prognostic value, some of which may serve as potential biomarkers for diagnosis and prognosis. In addition, the prognostic signatures constructed based on these prognostic RBPs can accurately predict the development of GC patients and have very good application prospects.

## Materials and methods

### Identification of differentially expressed RNA binding proteins (DERBPs)

We downloaded the original data containing primary gastric cancer. The 13 microarrays are all on the GPL570 platform, and the gastric cancer samples are all obtained by surgical resection. The data of each microarray is shown in Table 1. The RMA algorithm of the “affy” package is used to extract the expression data of each data set 19, and the “sva” package is used to remove batch effects and merge all data sets 20. The P value of the differentially expressed genes between the merged data was analyzed by the eBayes test of the “limma” package 21. The threshold for determining differentially expressed genes (DEGs) is set to |log2 fold change (FC)| ≥ 1.0 and false discovery rate (FDR) <0.05. Based on the RNA-binding protein database RBPTD (http://www.rbptd.com/) 22, we selected differentially expressed RNA-binding proteins (DERBPs) from these DEGs that met the screening criteria.

### Gene Ontology (GO) and Kyoto Encyclopedia of Genes and Genomes (KEGG) gene function enrichment analysis

The GO database has three categories, namely Biological Process (BP), Cellular Component (CC) and Molecular Function (MF). Each describes the molecular functions that gene products may perform. The cellular environment and the biological processes involved. The KEGG database helps to research genes and expression information as a whole network. KEGG integrates data on genomes, chemical molecules and biochemical systems, including metabolic pathways (PATHWAY), drugs (DRUG), diseases (DISEASE), gene sequences (GENES), and genomes (GENOME).

### Identify prognostic-related RBPs

In order to identify RBPs related to prognosis, we used a series of complete statistical analyses. We first use the “survival analysis” package to perform single-factor Cox regression analysis on the probe ID of DERBPs. Subsequently, based on the results of the previous step, Lasso regression was used for further screening to obtain RBPs that are significantly related to the prognosis of GC patients. In addition, we also used multivariate Cox regression analysis to test the results of Lasso regression and found hub RBPs that can independently predict the prognosis of GC.

### Construction and verification of the prognostic model of RBPs

We randomly divide all GC samples into training set (n=248) and validation set (n=244). We first used multivariate Cox regression analysis in the training set to construct a risk ratio model for predicting the prognosis of GC based on hub RBPs. We first used multivariate Cox regression analysis in the training set to construct a risk ratio model for predicting the prognosis of GC based on hub RBPs. We obtained the HR of hub RBPs, the 95% confidence interval of HR and the regression coefficient (β) of the corresponding gene. According to the expression of each gene and the regression coefficient, we can calculate the risk score of each patient according to: Risk score=β1*Exp1+β2*Exp2+βi*Expi. The GC patients were divided into high-risk and low-risk groups based on the median risk score, and Kaplan-Meier survival curve was drawn. The difference in OS between the two groups was analyzed by log-Rank test. We also draw a 5-year ROC curve with “SurvivalROC” package, and calculate the AUC value to evaluate the predictive ability of the predictive model. In addition, we also collected the clinical information of these chips (Supplementary Table 1), and evaluated the risk scores and clinicopathological characteristics of the HR value and P value through single factor and multivariate Cox regression analysis to determine whether the risk value is an independent prognostic factor for GC patients. In order to verify the signature we built, we not only used the validation set to verify the model built using the same method, but also conducted an overall analysis of all GC samples to verify the prognostic model of RBPs.

### Bioinformatics analysis

We used the chi-square test to analyze the relationship between the expression of each hub RBPs and the clinicopathological characteristics of patients with GC, and search for the potential mechanisms that affect tumor progression. In addition, in order to study the differences between different risk groups, we first analyzed the relationship between high and low risk groups and clinicopathological characteristics, and secondly, we also used Gene Set Enrichment Analysis (GSEA) to further analyze the overall differences between different risk groups 23. We use GSEA4.0 based on the molecular signature database (MSigDB), with hallmark7.1 as the control gene and |NSE|> 1, FDR <0.001 as the screening conditions to identify the development mechanism of GC in the high-risk group.

### RBPs nomogram construction and verification

We use “rms” to draw a nomogram based on the RBPs prognostic risk model, and obtain the corresponding score by analyzing the expression level of each hub RBPs, and adding the scores of all hub genes to obtain the corresponding total score. By drawing a vertical line on the total score line, we can predict the probability of survival for patients with GC in 1 to 5 years. In addition, in order to test the prediction ability of the nomogram, we draw a 5-year calibration curve by analyzing the survival probabilities of the predicted value and the actual value at the quartile of all GC patients. If the actual value is close to the predicted value, the nomogram has good predictive performance.

### Verification of hub RBPs expression and prognosis

In order to verify the prognosis of hub RBPs, we used Kaplan-Meier Plotter (http://kmplot.com/) to verify it in the GSE29272 data set 24. For the verification of hub RBPs expression, we first use the GEPIA network tool for verification, which contains data from the TCGA and GTEx databases 25. In addition, we also collected surgical samples from 10 pairs of gastric cancer patients. The process was approved by the patient's informed consent and the ethics committee of the Second Affiliated Hospital of Nanchang University. After homogenizing the clinical samples, the Trizol (Thermo Fisher, USA) method was used to extract total RNA. The obtained RNA was reverse transcribed using reverse transcription kit RR047A (Takara, Japan). ACTB was used as the internal reference gene, and the mRNA expression of hub RBPs was analyzed by fluorescence quantitative PCR using the RR820 kit (Takara, Japan) on the 7900-HT system (Thermo Fisher, USA). The primers used are all synthesized by Shanghai Shenggong Company, and the sequences of all primers are in Supplementary Table 2.

## Results

### Identify DERBPs

**Figure 1:**
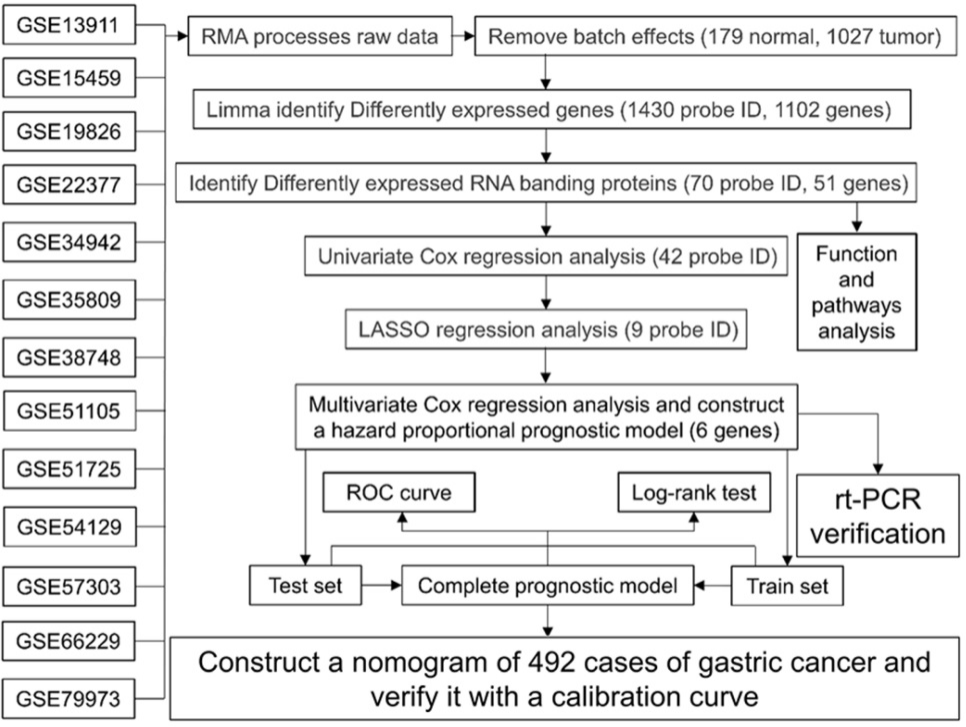
Work flow chart of this research.

The flowchart of this study is shown in Fig. 1, and the details of the GEO data set included in this study are shown in Table 1. After extracting expression data from each data set, removing batch effects and merging, a single expression data of 179 normal stomach and 1027 GC tissues were obtained. After the difference analysis of the “limma” package, we obtained 1430 probe differences (1102 genes) that met the screening conditions, and used R to draw the heat map and volcano map. After further screening, we got 70 One probe ID (51 genes) representing RBPs, of which 25 RBPs were up-regulated and 26 genes were down-regulated (Table 2).

### GO and KEGG gene function enrichment analysis

We performed GO and KEGG functional enrichment analysis on these DERBPs. The results suggest that 51 DERBPs are enriched in posttranscriptional regulation of gene expression, mRNA binding, nucleicase activity, regulation of cellular amide metabolic process, catalytic activity, acting on RNA, nuclear acid phosphodiester bond hydrolysis, RNA catabolic process, AU-rich element binding, RNA modification, debtinase activity, mRNA processing, regulation of mRNA metabolic process, DNA modification, ribonucleoprotein complex biogenesis (Table 3). For the KEGG functional enrichment analysis, we did not find an item that met the filter criteria.

### Identification of prognostic-related hub RBPs

**Figure 2:**
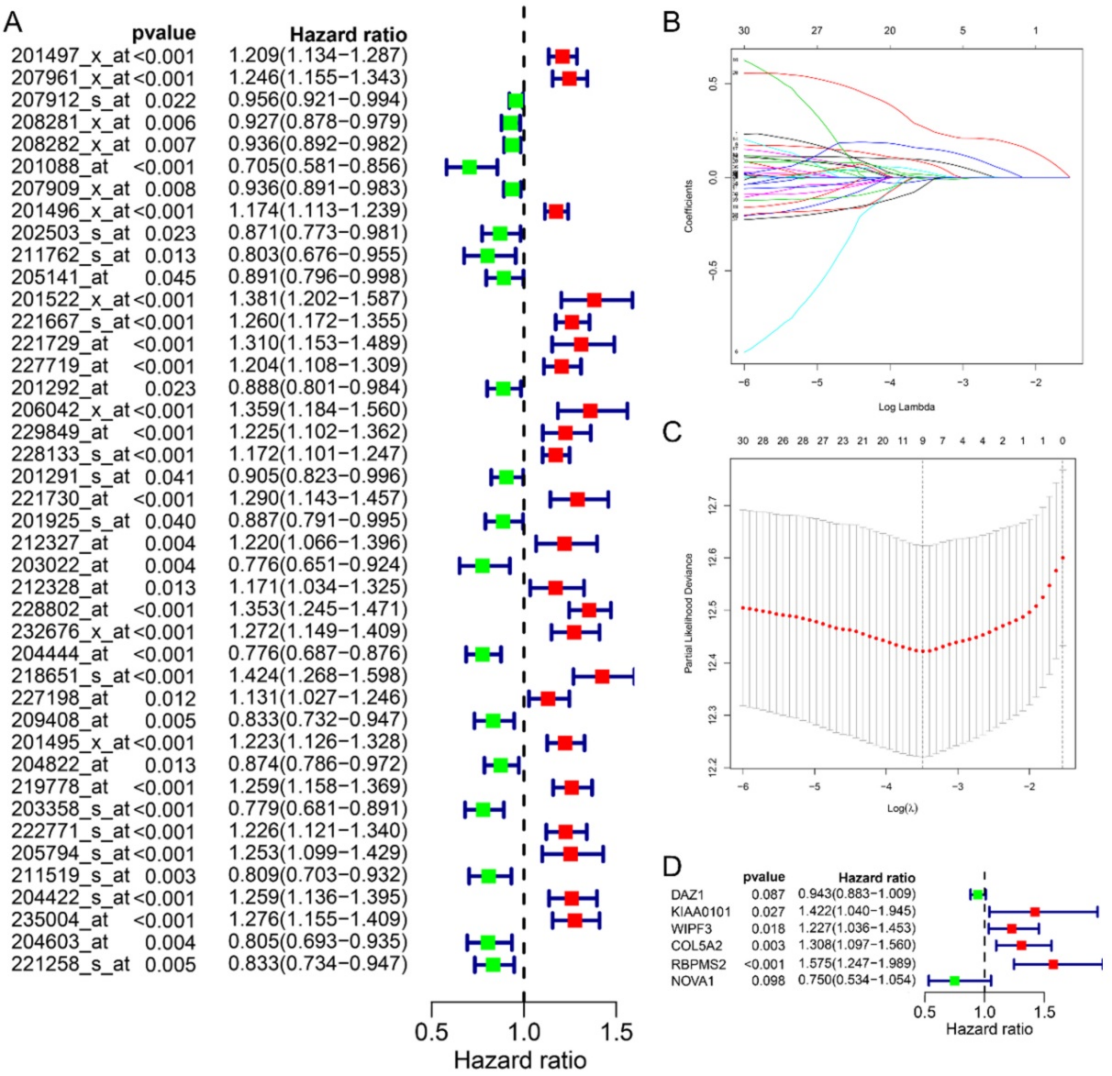
Identify prognostic-related hub RBPs. **A** Univariate Cox regression analysis identified prognostic-related RBPs in the entire cohort. **B** Selecting the best parameters for gastric cancer in LASSO regression analysis. **C** Multivariate Cox regression analysis identified prognostic-related hub RBPs.

In this study, we collected a total of 492 samples with clinical information for further research. First, the univariate Cox regression analysis was used to analyze the relationship between 70 RBPs probes and prognosis, and the results suggested that 42 RBPs probes were related to the prognosis of GC patients (Fig. 2A). Subsequently, Lasso regression further analyzed the results of the previous step and screened the RBPs probes that are significantly related to the prognosis. We obtained 9 key RBPs probes that are significantly related to the prognosis of GC patients (Fig. 2B and C). In addition, we also used multivariate Cox regression analysis of these 9 key probes, and the results showed that 6 hub RBPs probes (6 RBPs) are independent prognostic factors for patients with GC. Among them, HR>1 includes KIAA0101, WIPF3, COL5A2, RBPMS2, and HR<1 includes DAZ1 and NOVA1.

### Construction of a prognostic model of GC based on RBPs

**Figure 3:**
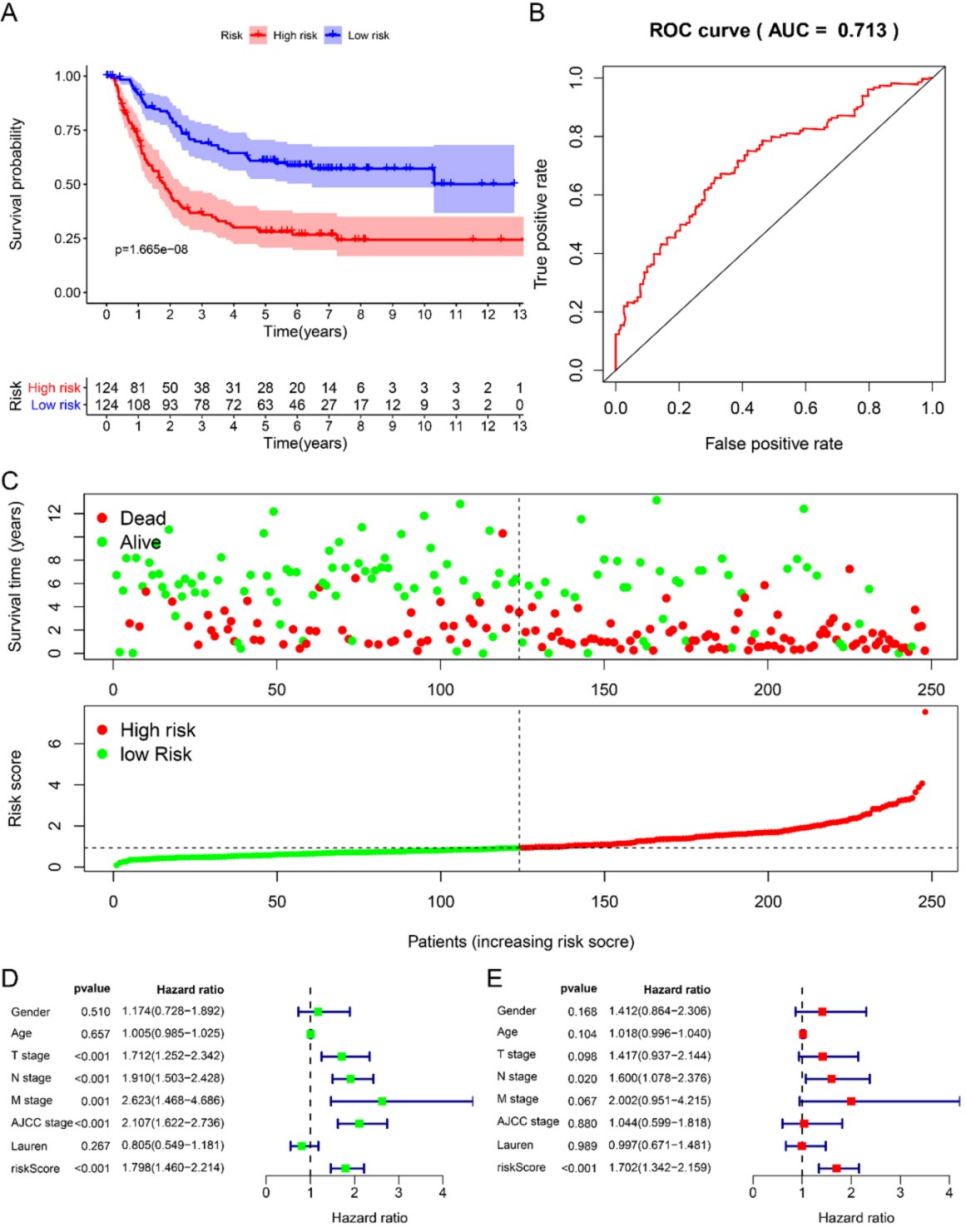
Prognostic analysis of 6-RBPs signature in the train cohort. **A** The survival analysis of 6-RBPs signature in the testing cohort. **B** 5-year time-dependent ROC for survival prediction models. **C** The distribution of risk score, OS and OS status of prognostic 6-RBPs signature in the testing cohort. **D** Univariate Cox regression analysis on the prognosis of clinicopathological characteristics and risk scores in patients with gastric cancer. **E** Multivariate Cox regression analysis on the prognosis of clinicopathological characteristics and risk scores in patients with gastric cancer.

We randomly divided 492 GC samples with clinical information into training set (n=248) and test set (n=244). We first assign the corresponding regression coefficients (β) to the 6 hub RBPs in the training set, and then synthesize their expression levels to construct a prognostic model. Each GC patient gets a risk score according to the following formula: Risk score= -0.058 *ExpDAZ1+ 0.3524 *ExpKIAA0101+ 0.2045 *ExpWIPF3 + 0.2687 *ExpCOL5A2 + 0.4544 * EXPRBPMS2 + -0.2872 * ExpNOV1, divided into high-risk group and low-risk group based on the median Risk group. The KM curve indicated that the survival rate of GC patients in the high-risk group was significantly lower than that in the low-risk group (Fig. 3A), and the 5-year AUC was calculated as 0.713 (Fig. 3B). The distribution of survival status indicated that the survival time and number of patients in the high-risk group was lower in the risk group, low (Fig. 3C).

**Figure 4:**
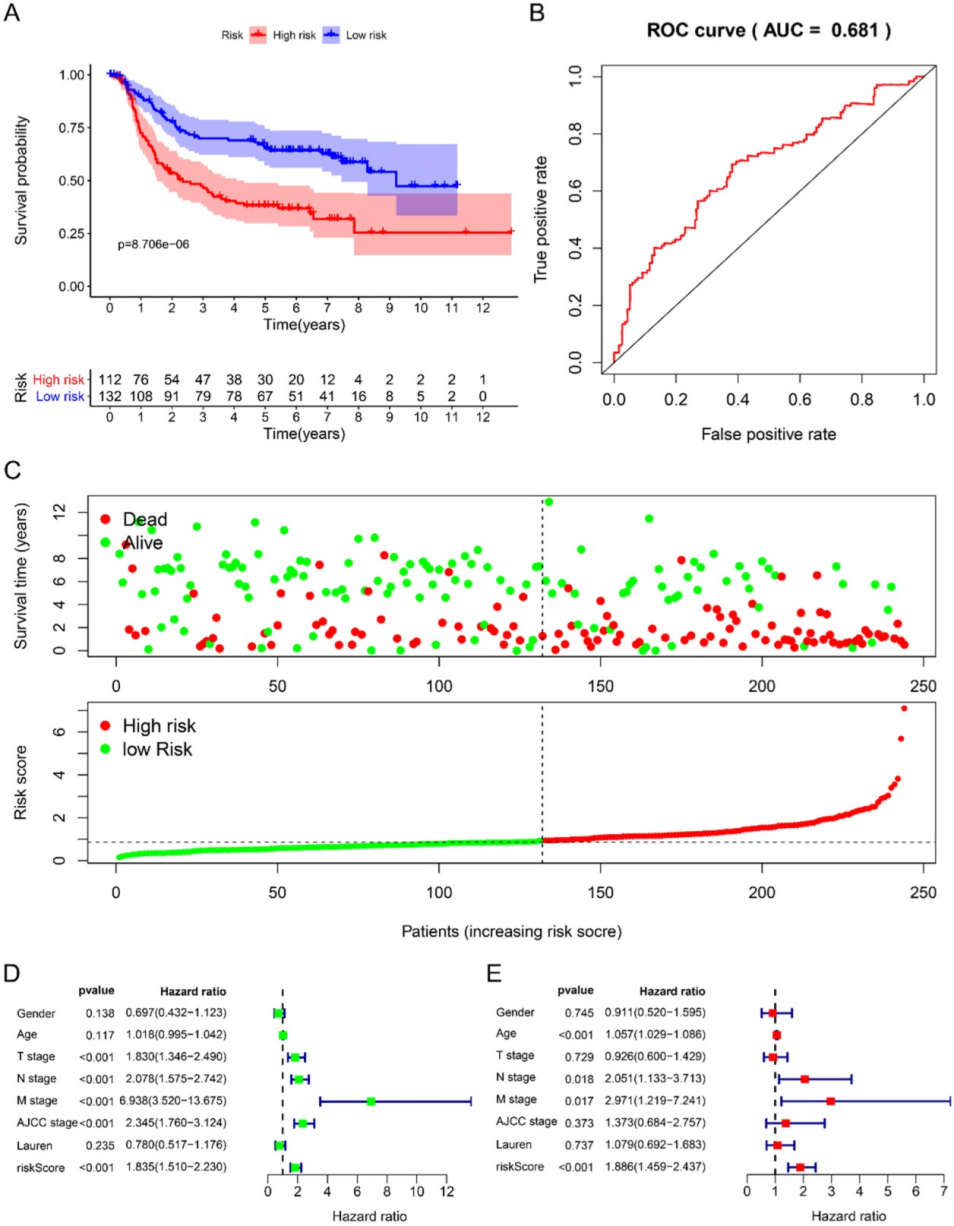
Prognostic analysis of 6-RBPs signature in the test cohort. **A** The survival analysis of 6-RBPs signature in the testing cohort. **B** 5-year time-dependent ROC for survival prediction models. **C** The distribution of risk score, OS and OS status of prognostic 6-RBPs signature in the testing cohort. **D** Univariate Cox regression analysis on the prognosis of clinicopathological characteristics and risk scores in patients with gastric cancer. **E** Multivariate Cox regression analysis on the prognosis of clinicopathological characteristics and risk scores in patients with gastric cancer.

**Figure 5:**
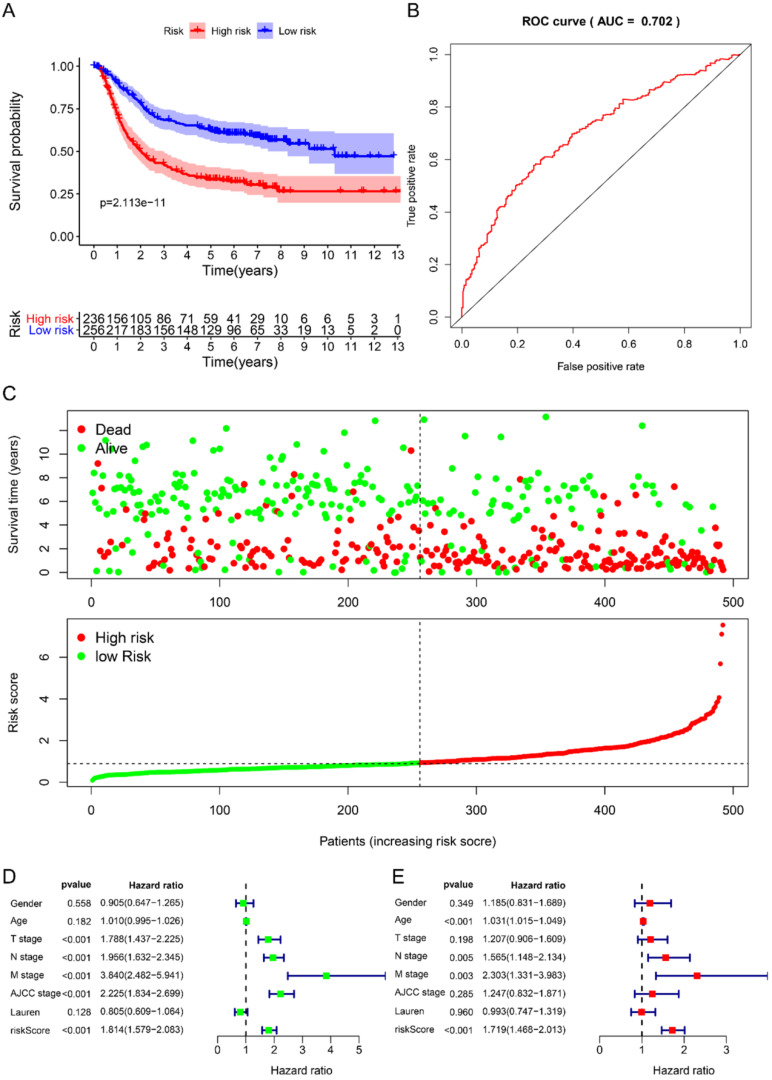
Prognostic analysis of 6-RBPs signature in the complete cohort. **A** The survival analysis of 6-RBPs signature in the testing cohort. **B** 5-year time-dependent ROC for survival prediction models. **C** The distribution of risk score, OS and OS status of prognostic 6-RBPs signature in the testing cohort. **D** Univariate Cox regression analysis on the prognosis of clinicopathological characteristics and risk scores in patients with gastric cancer. **E** Multivariate Cox regression analysis on the prognosis of clinicopathological characteristics and risk scores in patients with gastric cancer.

Using univariate Cox to analyze the relationship between risk value and clinicopathological parameters and overall survival (Fig. 3D), T stage [HR=1.712, 95%CI (1.252-2.342)], N stage [HR=1.910, 95%CI (1.503-2.428)], AJCC stage [HR=2.107, 95%CI (1.622-2.736)] and risk value [HR=1.798, 95%CI (1.460-2.214)] are related to prognosis. Multivariate Cox regression analysis further showed (Fig. 3E) that N stage [HR=1.600, 95%CI (1.078-2.376)] and risk score [HR=1.702, 95%CI (1.342-2.159)] are independent of GC patient prognostic factors. There are 112 samples in the high-risk group in the training set and 132 samples in the low-risk group. The KM curve indicates that the high-risk group has a worse prognosis (Fig. 4A), 5-year AUC=0.681 (Fig. 4B), and the survival status diagram is similar to the training set (Fig. 4C). Single-factor Cox regression analysis shows that the risk value of T stage, N stage, M stage, AJCC stage is related to OS (Fig. 4D). Multivariate Cox regression shows that Age, N stage, M stage, and risk values can independently predict the prognosis of GC patients (Fig. 4E). In addition, we combined the training set and the validation set to form a complete prognostic model. The K-M curve showed that the low-risk group had a better prognosis (Fig. 5A), 5-year AUC=0.702 (Fig. 5B), and the survival status chart indicated that the high-risk group had lower survival time and number of survivals (Fig. 5C). Single-factor Cox regression analysis shows that T stage, N stage, M stage, AJCC stage, and risk scores are related to prognosis (Fig. 5D). Multivariate Cox regression analysis suggests that Age, N stage, M stage, and risk values have the ability to independently predict GC (Fig. 5E).

### Bioinformatics analysis

**Figure 6:**
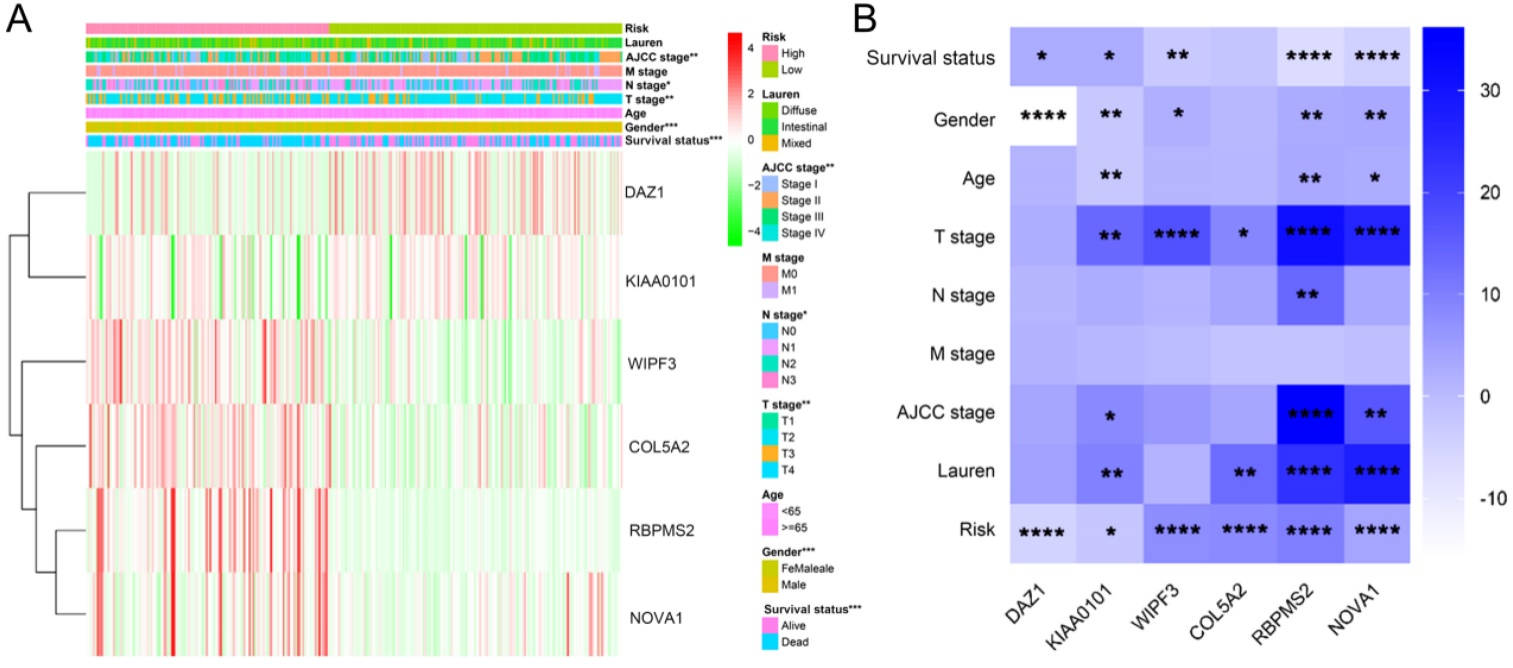
The relationship between different risk groups and 6hub-RBPs and clinicopathological characteristics. **A** The relationship between different risk groups and 6hub-RBPs and clinicopathological characteristics. **B** The relationship between 6-hub RBPs and clinicopathological characteristics.

**Figure 7:**
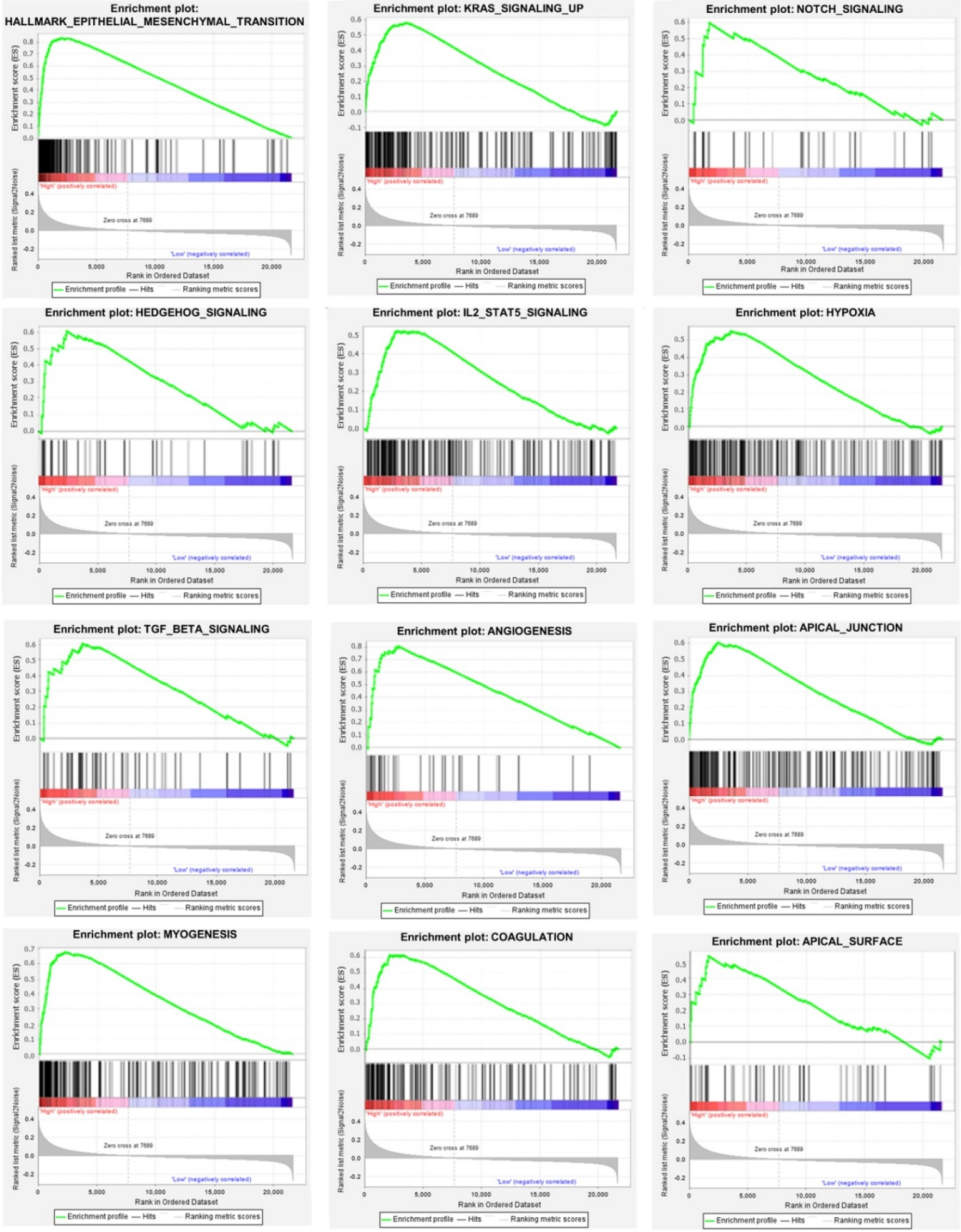
Gene set enrichment analysis of functional gene set differences between high and low risk groups.

We used the chi-square test to analyze the clinicopathological characteristics and hub RBPs (Fig. 6A), with different risk groups. The results show that AJCC stage, N stage, T stage, Gende and survival status are related to high and low risk values. Among the 6 hub RBPs, RBPMS2, KIAA0101 and NOVA1 are related to 6 or more clinicopathological features, while WIPF3, COL5A2 and DAZ1 are only related to a few clinicopathological features (Fig. 6B). In addition, in order to study the differences between the high and low risk groups of GC patients, we used GSEA to analyze them. The results indicate (Fig. 7) that the samples of the high-risk group were mostly enriched in epithelial-mesenchymal transition, KRAS signal up-regulation, NOTCH signal, TGF-β signal, Angiogenesis, Hedgehog signal, IL2/STAT5 signal, Hypoxia, Myogenesis, Coagulation, UV response UP, Apical junction, APICAL SURFACE.

### Nomogram drawing and verification

**Figure 8:**
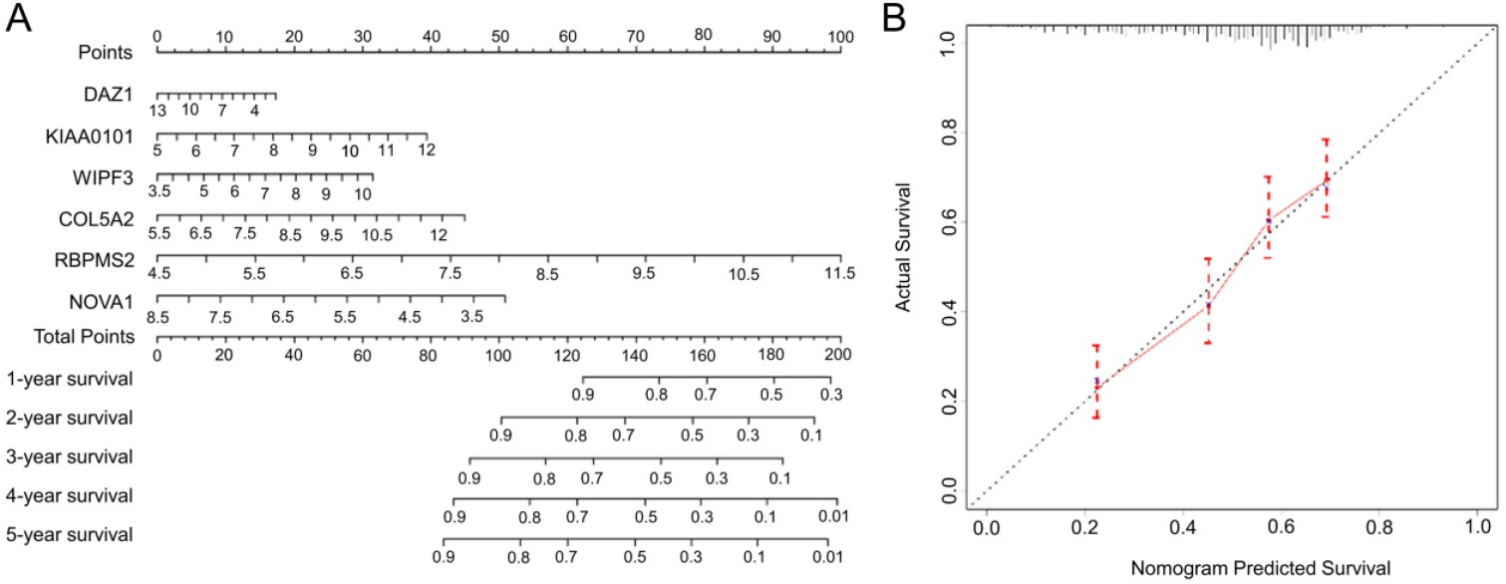
The nomogram and the calibration chart are used to predict the OS survival rate of gastric cancer patients. **A** Nomogram for the prediction of OS at 1-5 year. **B** Calibration plots for predicting OS at 5 year.

Based on the complete prognosis model we constructed, we established a nomogram to predict the survival rate of GC in 1-5 years (Fig. 8A). By detecting the expression levels of the 6 hub RBPs and obtaining the corresponding scores, the total scores obtained can intuitively predict the short-term survival probability of patients with GC, which will provide clinicians with precise and individualized diagnosis and treatment. In order to verify the accuracy of the nomogram, we calculated the predicted survival rate of 492 samples as the X-axis and the actual survival rate as the Y-axis to draw a 5-year calibration curve. The results indicate (Fig. 8B) that the predicted survival rate of the nomogram is almost equal to the actual survival rate, and the slope of the drawn curve is almost 1, indicating the accurate predictive ability of the nomogram.

### Expression and prognostic verification of hub RBPs

**Figure 9:**
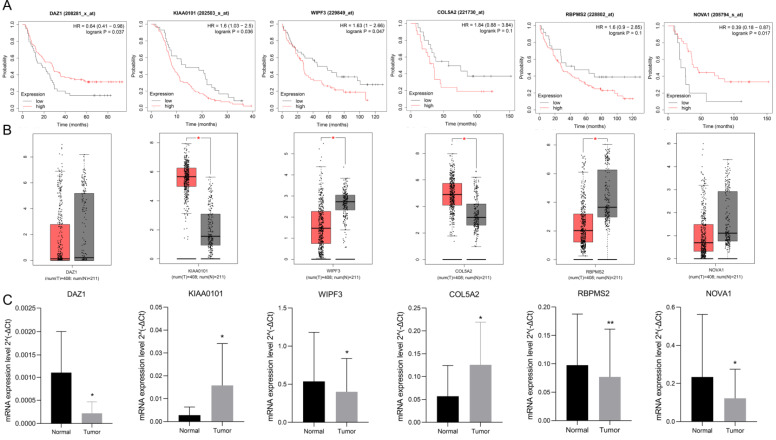
Verify the prognosis and expression of 6 hub-RBPs. **A** K-M plotter verifies the prognosis of 6 hub-RBPs. **B** GEPIA verifies the expression level. **C** rt-PCR verifies the expression of hub RBPs in clinical samples.

Use K-M plotter to analyze 6 hub gene prognosis prompts (Fig. 9A), high expression of DAZ1, NOVA1 suggests a better prognosis (P<0.05), increased expression of KIAA0101, WIPF3 prompts a poor prognosis (P<0.05), high expression of COL5A2, RBPMS2 It is nega-tively correlated with prognosis, but it is not statistically significant (P=0.1). We used GEPIA to verify the expression of 6 hub RBPs, which contains the se-quencing data of TCGA and GTEx. GEPIA analysis results showed (Fig. 9B) that the expression of KIAA0101 and COL5A2 in GC tissues was significantly higher than that in normal tissues (P<0.05), and the expression of WIPF3 and RBPMS2 in normal tissues were increased (P<0.05). It is worth noting that DAZ1 and NOVA1 were higher in normal tissues, but they were not statistically significant. rt-PCR verified the expression of hubRBPs in 10 pairs of clinical samples. The results showed that (Fig. 9C) DAZ1, WIPF3, RBPMS2, and NOVA1 were low expressed in gastric cancer (P<0.05), and KIAA0101 and COL5A2 were highly expressed (P<0.05).

## Discussion

RBPs have diverse structures and functions, which regulate several necessary cellular processes. Some of these RBPs are usually expressed and conservatively evolved to maintain the basic processes of the cell. Unregulated expression of RNAs can cause many diseases, including cancer 26. The abnormality of RBPs has an important influence on tumor phenotype. Such as, mutations in TRBP lead to abnormal expression of miRNA and cancer cell proliferation and differentiation 27. hnRNPs promote the synthesis of PKM2, thereby enhancing the Warburg effect 28. RBM38 can inhibit breast cancer metastasis by promoting STARD13-related competitive endogenous RNA network 29. In GC, only a few literatures have reported that RBPs are dysregulated in GC, there are also reports that RBPs are involved in tumor cell proliferation, metastasis and other phenotypes 16-18. To this end, we collected 13 GPL570 platform microarray data in the GEO database to systematically identify the differentially expressed RBPs in GC. We have identified 25 up-regulated and 26 down-regulated RBPs. These DERBPs have been reported to be abnormally expressed in GC and play an important role. Among them, EZH2 is up-regulated in GC and is a good prognostic marker, which can inhibit p21 and promote the proliferation of GC 30. In addition, it can also inhibit FBXO32 epigenetically and contribute to the resistance of GC 31. However, some studies suggest that it plays a tumor suppressor effect in GC. Research by Zhao et al. suggested that EZH2-mediated epigenetic suppression of EphB3 can down-regulate E-cadherin and vimentin and inhibit GC metastasis 32. This indicates that the role of RBPs in tumors is heterogeneous.

In order to further understand the possible mechanism of DERBPs affecting the progression of GC, we performed enrichment analysis on 51 RBPs. The results show that these DERBPs are mainly enriched in items such as posttranscriptional regulation of gene expression, mRNA binding, RNA catabolic process, RNA modification, and mRNA processing, some of them have been considered to play an important role in cancer progression. The most important thing in these processes is that RBPs can bind to mRNA, promote its stability, and regulate target gene expression. For example, PTBP3 can regulate the expression of the transcription factor ZEB1 by binding to the 3'UTR of its mRNA, thereby preventing its degradation, inducing the epithelial-mesenchymal transition of breast tumor cells and promoting their invasive growth and metastasis 33. In addition, RBPs can also promote the degradation of mRNA. IGF2BP3 can accelerate the degradation of EIF4E-BP2 mRNA, thereby promoting the proliferation of cervical cancer cells 34. RBP-mediated selective splicing is a post-transcriptional regulatory mechanism that contributes to protein diversity and mRNA stability. Abnormal or wrong splicing is the main cause of abnormal function of RBPs, which promotes cancer 35. Research by Liu et al. showed that SNRPB, the core component of alternative splicing, regulates the alternative splicing of intron 7 and its expression in RAB26 mRNA by activating NMD, mediating tumor growth and metastasis 36. RBPs also participate in the addition of a poly(A) tail to the 3'end of the mRNA. Among them, alternative polyadenylation is a widespread basic regulatory mechanism in eukaryotes. This process can promote mRNA maturation, stability, nuclear transport and efficient translation. In cancer, research suggests that RBPs can regulate polyadenylation of transcripts, extend poly(A) tails and improve translation efficiency of target genes, and promote tumor progression 37.

In addition, RBPs can not only affect tumor occurrence and development, but also serve as prognostic markers. We performed univariate Cox regression, Lasso regression and multivariate Cox regression analysis on all RBPs probe probes, and obtained 4 RBPs (DAZ1, KIAA0101, WIPF3, COL5A2, RBPMS2, NOVA1) that independently predict the prognosis of GC. In addition, we also analyzed their relationship with clinicopathological characteristics, suggesting that they are closely related to T staging and Lauren classification. There are reports that GC patients with high expression of KIAA0101 showed a high recurrence rate, and accompanied by a poor prognosis, the vitality of GC cells was significantly inhibited after inhibiting its expression 38. Research by Li et al. suggested that the low expression of NOVA1 in GC is related to lymphatic metastasis and poor prognosis 39. Inhibiting the expression of NOVA1 in cells promotes the epithelial-mesenchymal transition of GC cells 39, 40. Although some genes have not been studied in GC, they have been reported in other tumors. Research by Hapkova et al. suggested that RBPMS2 is highly expressed in gastrointestinal stromal tumors, and indicated that it is a new diagnostic marker and a potential cancer treatment target 41. Among them, some reports are different from our conclusions. COL5A2 is considered to be a favorable prognostic factor for tongue squamous cell carcinoma 42. However, high expression of this gene in muscle-invasive bladder cancer suggests that the prognosis is poor, and it is closely related to tumor invasion 43, 44. DAZ1 and WIPF3 have not been reported in tumors, which suggests that they may be new predictive markers and therapeutic targets for GC and require more in-depth research.

Our other result is the construction of a prognostic signature based on RBPs. First we divide the whole cohort into a train set and a test set, build a prognostic signature in the train set based on hub RBPs, and verify it in the test set. The results show that the survival rates of the two concentrated high-risk groups are lower than those of the low-risk group, and the risk scores obtained are independent prognostic factors. The 5-year AUC calculated by the ROC curve is not 0.713 and 0.681, respectively, indicating that the model has broad applicability and accuracy. We also integrated the two sets into a complete prognostic signature, and obtained the same result as the training set after analysis, with a 5-year AUC value of 0.702. We further used the chi-square test to analyze the relationship between clinicopathological characteristics between high and low risk groups. The results show that it is related to AJCC stage, N stage, T stage, gender and survival status. We also used GSEA to analyze the differences in mechanisms affecting GC between high and low risk groups. The results suggest that high-group GC samples are mainly enriched in epithelial-mesenchymal transition (EMT), Kras, Notch, TGF-β, angiogenesis, Hedgehog, IL2-Stat5, hypoxia and other signals. Multiple reports suggest that RBPs mediate the above related signals to affect tumor progression. EMT is a biological process in which differentiated epithelial cells lose their epithelial characteristics and gain mesenchymal cell migration. Tumor cells with this characteristic exhibit high invasiveness and antagonistic ability to radiotherapy and chemotherapy. There are many ways to participate in EMT, including Notch, TGF-β, Hedgehog, Wnt, etc. 45. It is reported that KIAA0101 can participate in the EMT of liver cancer and induce tumor cell migration and angiogenesis 46. This may be triggered by the activation of Wnt signal conduction 47-49. NOVA1 can also participate in EMT transition, which may be triggered by direct binding to β-catenin RNA 50. Other ways have played an indispensable role in tumor development, so won't repeat them.

We used the nomogram to optimize the prognostic signature and visualized it. Clinicians can detect the expression of hub RBPs and obtain the corresponding total score, which can predict the survival rate of GC patients in 1-5 years. In addition, we drew a calibration chart. Through the calibration chart, we can find that the predicted 5-year survival rate is almost the same as the actual survival rate, which shows the precise predictive ability of the nomogram. To verify the expression and prognosis of hub-RBPs. We verified the expression of hub RBPs in GEPIA, suggesting that their expression trends are consistent with our analysis. The results of K-M plotter suggest that the predictive ability of hub RBPs for GC is consistent with our analysis.

In a word, we have integrated 13 microarrays on the GPL570 platform, and after systematic bioinformatics analysis, we have identified the differentially expressed RBPs in GC and analyzed their potential mechanisms affecting GC. We also constructed a GC prognostic signature based on RBPs, analyzed and verified its good predictive abilityWe also verified the expression of hub RBPs that construct the prognostic signature through external databases and clinical samples. We found that some RBPs are related to the pathogenesis of GC, but there is a lack of relevant literature reports and further studies on GC. This is the limitation of our research.

## Supplementary Material

Supplementary tables.

## Figures and Tables

**Table 1:** GEO data sets included in this study

GEO datasets	Year	Country	Platform	Sample	Tumor (n)	Normal (n)
GSE66229	2015	USA	GPL570	GC/Normal	300	100
GSE54129	2017	China	GPL570	GC/Normal	111	21
GSE13911	2008	Italy	GPL570	GC/Normal	38	31
GSE19826	2010	China	GPL570	GC/Normal	12	15
GSE79973	2016	China	GPL570	GC/Normal	10	10
GSE51725	2013	Japan	GPL570	GC/Normal	8	2
GSE15459	2009	Switzerland	GPL570	GC	200	0
GSE51105	2014	Australia	GPL570	GC	94	0
GSE35809	2012	Singapore	GPL570	GC	70	0
GSE57303	2014	China	GPL570	GC	70	0
GSE34942	2014	Singapore	GPL570	GC	56	0
GSE22377	2011	Germany	GPL570	GC	43	0
GSE38749	2012	Brazil	GPL570	GC	15	0

**Table 2:** 51 differentially expressed RNA binding proteins

Upregulate RNA binding protein				Down-regulate RNA binding protein			
Prob ID	Gene ID	Log2FC	P	Prob ID	Gene ID	Log2FC	P
203820_s_at	IGF2BP3	2.83	1.46E-68	225939_at	EIF4E3	-1.03	9.43E-64
203819_s_at	IGF2BP3	2.62	1.52E-61	235004_at	RBM24	-1.03	8.64E-27
201291_s_at	TOP2A	2.06	1.60E-72	204422_s_at	FGF2	-1.04	6.92E-28
201292_at	TOP2A	1.87	4.50E-68	219778_at	ZFPM2	-1.06	1.64E-19
209408_at	KIF2C	1.65	9.88E-89	232676_x_at	MYEF2	-1.06	1.08E-28
211519_s_at	KIF2C	1.21	5.58E-63	222771_s_at	MYEF2	-1.21	6.44E-29
204822_at	TTK	1.62	2.78E-54	212328_at	LIMCH1	-1.07	6.54E-42
202870_s_at	CDC20	1.61	2.41E-64	212327_at	LIMCH1	-1.10	6.49E-50
223229_at	UBE2T	1.55	1.17E-76	208281_x_at	DAZ1	-1.09	6.49E-08
203358_s_at	EZH2	1.53	4.76E-76	208282_x_at	DAZ1	-1.32	4.91E-09
221258_s_at	KIF18A	1.42	8.18E-64	207909_x_at	DAZ1	-1.33	2.36E-09
204603_at	EXO1	1.40	5.88E-87	207912_s_at	DAZ1	-1.78	8.96E-11
221730_at	COL5A2	1.39	1.21E-65	205141_at	ANG	-1.09	3.31E-36
221729_at	COL5A2	1.36	1.16E-67	239587_at	TLR3	-1.09	1.79E-35
225827_at	AGO2	1.33	2.04E-119	206042_x_at	SNRPN	-1.11	1.10E-48
213310_at	AGO2	1.12	1.45E-74	201522_x_at	SNRPN	-1.15	2.00E-51
211762_s_at	KPNA2	1.32	5.99E-93	218651_s_at	LARP6	-1.15	4.93E-43
201088_at	KPNA2	1.11	2.68E-86	207158_at	APOBEC1	-1.19	2.60E-22
204444_at	KIF11	1.28	4.36E-47	213397_x_at	RNASE4	-1.20	1.86E-49
201926_s_at	CD55	1.27	2.67E-41	205158_at	RNASE4	-1.23	2.58E-43
1555950_a_at	CD55	1.24	8.41E-41	56256_at	SIDT2	-1.20	2.36E-104
201925_s_at	CD55	1.20	1.92E-38	205794_s_at	NOVA1	-1.23	2.71E-52
202503_s_at	KIAA0101	1.23	2.11E-41	201495_x_at	MYH11	-1.23	1.40E-25
218984_at	PUS7	1.20	3.75E-90	207961_x_at	MYH11	-1.77	2.79E-41
206632_s_at	APOBEC3B	1.15	6.95E-22	228133_s_at	MYH11	-1.89	4.34E-34
205895_s_at	NOLC1	1.14	1.08E-98	201497_x_at	MYH11	-2.15	3.31E-41
214697_s_at	PTBP3	1.12	3.86E-56	201496_x_at	MYH11	-2.44	2.35E-40
201614_s_at	RUVBL1	1.10	1.36E-101	209309_at	AZGP1	-1.46	2.04E-22
203612_at	BYSL	1.08	3.49E-87	221868_at	PAIP2B	-1.57	1.20E-111
213175_s_at	SNRPB	1.07	1.04E-93	206160_at	APOBEC2	-1.60	1.09E-102
203022_at	RNASEH2A	1.05	1.33E-68	228802_at	RBPMS2	-1.65	5.45E-51
225841_at	HENMT1	1.04	5.88E-49	229849_at	WIPF3	-1.66	1.14E-64
218239_s_at	GTPBP4	1.00	6.11E-82	221667_s_at	HSPB8	-1.66	8.68E-38
-	-	-	-	227719_at	SMAD9	-1.76	2.84E-50
-	-	-	-	201785_at	RNASE1	-1.91	1.43E-87
-	-	-	-	227198_at	AFF3	-1.92	3.17E-74
-	-	-	-	205200_at	EXOSC7	-2.21	1.62E-98

**Table 3:** Gene Ontology enrichment analysis results of differentially expressed RBPs

Accession	Ontology	Description	*P*-value	FDR
GO:0010608	BP	posttranscriptional regulation of gene expression	1.62E-10	2.11E-07
GO:0034248	BP	regulation of cellular amide metabolic process	2.51E-07	8.20E-05
GO:0090305	BP	nucleic acid phosphodiester bond hydrolysis	2.78E-06	6.04E-04
GO:0006401	BP	RNA catabolic process	9.62E-06	1.79E-03
GO:0009451	BP	RNA modification	1.09E-04	1.43E-02
GO:0006397	BP	mRNA processing	1.21E-04	1.43E-02
GO:1903311	BP	regulation of mRNA metabolic process	1.71E-04	1.86E-02
GO:0006304	BP	DNA modification	3.43E-04	3.44E-02
GO:0022613	BP	ribonucleoprotein complex biogenesis	4.07E-04	3.79E-02
GO:0003729	MF	mRNA binding	3.75E-08	2.45E-05
GO:0004518	MF	nuclease activity	2.14E-07	8.20E-05
GO:0140098	MF	catalytic activity, acting on RNA	1.17E-06	3.06E-04
GO:0017091	MF	AU-rich element binding	8.90E-05	1.43E-02
GO:0019239	MF	deaminase activity	1.10E-04	1.43E-02
