# Supplementary material for: Integrating 13 Microarrays to Construct a 6 RNA-binding proteins Prognostic Signature for Gastric Cancer patients
Source: J Cancer. 2021 Jun 11;12(16):4971–84. doi: 10.7150/jca.57225 (PMC8247375; doi:10.7150/jca.57225)
Supplement: Supplementary file 1 — Supplementary tables. [file jcav12p4971s1.pdf]

Supplementary Table 1 Clinicopathological information of patients with gastric cancer

Clinicopathological characteristics		Number (n)	Proportion
Gender	Female	101	33.6667
	Male	199	66.3333
Age	<60	106	35.3333
	>=60	194	64.6667
T stage	T1	2	0.6667
	T2	186	62.0000
	T3	91	30.3333
	T4	21	7.0000
N stage	N0	38	12.6667
	N1	131	43.6666
	N2	80	26.6667
	N3	51	17.0000
M stage	M0	273	91. 0000
	M1	27	9. 0000
AJCC stage	Stage I	30	10. 0000
	Stage II	97	32.3333
	Stage III	96	32, 0000
	Stage IV	77	25.6667
Lauren classficition	Diffuse	135	45, 0000
	Intestinal	146	48.6667
	Mixed	19	6.3333

Supplementary Table 2 Hub RBPs primer sequence

Gene ID	Forward primer sequence (5' - 3')	Reverse Primer sequence (5' - 3')
DAZ1	AAGCCGTGGAATGGTAGCAA	AGCCATTGAAAGAAGGGCCA
KIAA0101	GTGCTTGGTTCTTCCACCTCTG	CCTTTTTGCCACTTGGGAGTTGG
WIPF3	CTCAGGGAAAGCCATTTGGT	GAAGCCCCCTTTTGTCACAC
COL5A2	AGCTGGGACCATCCCAAAAG	CCACTGACATGACAAAAGCGT
RBPMS2	CTGAACGGTATTCGCTTTGATCC	GTCCCGTGCGATGAAGTGT
NOVA1	TACTGAGCGAGTGTGCTTGAT	GTCTGGGGTTGTAGAATGCTG
